# Application of NRS2002 in Preoperative Nutritional Screening for Patients with Liver Cancer

**DOI:** 10.1155/2021/8943353

**Published:** 2021-08-27

**Authors:** Suling Huang, Shijie Wang, Yuankang Xie, Xiao He, Xiuying Yi, Jianhong Zhang, Zuomei Deng, Ling Yin

**Affiliations:** ^1^Department of Hepatological Surgery, First Affiliated Hospital of Gannan Medical University, Ganzhou 341000, Jiangxi Province, China; ^2^Department of Pharmacy, First Affiliated Hospital of Gannan Medical University, Ganzhou 341000, Jiangxi Province, China; ^3^Department of Thyroid Hernia Surgery, First Affiliated Hospital of Gannan Medical University, Ganzhou 341000, Jiangxi Province, China

## Abstract

**Objective:**

To explore the application of NRS2002 in preoperative nutritional screening of patients with liver cancer (LC).

**Methods:**

60 LC patients treated in the First Affiliated Hospital of Gannan Medical University (January 2018–May 2021) were chosen as the research objects, and split into group J without nutritional risk and group Q with nutritional risk according to the results of NRS2002 to compare the preoperative situation, surgery-related indexes, hematological indexes, postoperative recovery, and incidence of complications between the two groups.

**Results:**

Group J (*n* = 28) and group Q (*n* = 32) showed no obvious difference in preoperative situation, and patients' liver function indexes were within the normal range. The duration of surgery in group J was notably shorter compared with group Q (*P* < 0.05). Alanine aminotransferase (ALT), aspartate aminotransferase (AST), direct bilirubin (DBIL), and albumin in group J were notably different from those of group Q (*P* < 0.001) at 1 day after surgery. ALT and AST in group J were notably different from those of group Q at 3 days after surgery (*P* < 0.001). No obvious differences were observed in the hematological indexes between the two groups at 5 days after surgery (*P* > 0.05). The total amount of albumin infusion, postoperative hospitalization time, and hospitalization cost in group J were notably lower compared with group Q (*P* < 0.001). The incidence of complications in group J was notably lower compared with group Q (*P* < 0.05).

**Conclusion:**

Postoperative recovery of LC patients is closely related to their preoperative nutritional status, and those with poor nutritional status have a high incidence of postoperative complications and long recovery time. NRS2002 can effectively screen the nutritional status of patients and provide reference for prognosis evaluation.

## 1. Introduction

Liver cancer (LC) is a common clinical disease. According to the data of International Agency for Research on Cancer (IARC), the number of LC cases in China accounts for 55% of the total cases worldwide, and about 70% of the patients are in the middle and late stages when diagnosed, with an increasing annual mortality rate, endangering the life and health of Chinese residents. At present, surgical treatment is generally performed in practice to radically treat it. However, LC patients are often complicated with other diseases such as liver cirrhosis, their liver cells are seriously damaged, their metabolic function is reduced, and the inactivated function of steroid hormones is weakened, which jointly lead to the nutritional and metabolic imbalances in patients. Therefore, the possibility of malnutrition in patients with liver cancer during perioperative period reaches 80%, and severe malnutrition will increase the probability of surgical complications, enhance the adverse reactions of radiotherapy and chemotherapy, and affect the prognosis of patients [1–3]. In recent years, precise requirements have been put forward for surgical procedures. Preoperative assessment has become an integral part of surgical procedures, including liver function classification and imaging examination in LC patients. However, there were few preoperative examinations related to nutritional assessment. Since clinical practice shows that there is a close relationship between the nutritional status of patients and their postoperative recovery, it is extremely important to add preoperative nutritional assessment to the scope of preoperative examination [4–6]. NRS2002 is a common nutritional risk-screening scale in clinical practice, which can be applied in various types of hospitalized patients. Based on this, this paper aims to explore the application of NRS2002 in preoperative nutritional screening of LC patients, summarized as follows.

## 2. Materials and Methods

### 2.1. Preoperative Assessment

Sixty LC patients treated in the First Affiliated Hospital of Gannan Medical University (January 2018–May 2021) were chosen as the research objects and underwent preoperative evaluation, with steps as follows. (1) All the patients received routine examination. (2) The patients received indocyanine green excretive test with 15-minute retention and other methods to evaluate their liver function [7, 8]. (3) The patients underwent imaging examinations, including MRI examination and ultrasound examination to observe the tumor size, location, and other information and to determine whether extrahepatic metastasis occurred.

This study was in line with the principles of Declaration of Helsinki and was approved by the Ethics Committee of the First Affiliated Hospital of Gannan Medical University.

### 2.2. Inclusion Criteria

(1) The patients or their families fully recognized the study process and signed the informed consent. (2) The patients were diagnosed with LC after examination, and the lesions could be removed by hepatectomy. (3) The liver function of patients was graded as A [9, 10]. (4) The 15-minute retention rate of indocyanine green excretion test was 20% and below.

### 2.3. Exclusion Criteria

(1) The patients had mental problems or could not communicate with others. (2) The patients had other organic diseases. (3) The patients had liver function grade below A. (4) The patients had extrahepatic metastasis of cancer or tumor thrombus in the portal vein and primary branches. (5) The patients received radiotherapy and chemotherapy before surgery. (6) The patients quit the study halfway.

### 2.4. Methods

#### 2.4.1. Surgical Methods

(1) All surgeries of the patients were operated by the same surgical group. The surgical plans were selected according to the actual situation of the patients, and the ultrasound-assisted technology was adopted to clarify the sites for resection. (2) Portal triad clamping (Pringle) was selected for hepatic inflow occlusion. The liver tissue was separated by the fine clamp method, and the pipeline structure of the section was sutured, except for the liver section.

#### 2.4.2. Perioperative Nutrition Support Schemes

(1) The patient did not take nutritional support before surgery and continued to follow the routine dietary. (2) After surgery, enteral nutrition support was given to the patients. The patients could drink water at 12 h after surgery. With low amount and high frequency, the patients drank enteral nutrient solution (Nutricia Pharmaceutical Co., Ltd., Wuxi Branch; NMPA approval no. H20030012) at 24 h after surgery with 500 ml each day and was increased to 1000 mL within 3 days, accompanied by semiliquid diet.

### 2.5. Observation Criteria

1. Grouping of patients: the patients were split into group J without nutritional risk (NRS2002 score <3 points) and group Q with nutritional risk (NRS2002 score ≥3 points) according to the results of NRS2002, and the number of each group was counted [11–14].
2. Preoperative situation: the comparison items included gender, age, underlying diseases, alanine aminotransferase (ALT) level, aspartate aminotransferase (AST) level, total bilirubin (TBIL) level, direct bilirubin (DBIL) level, and albumin level.
3. Surgery-related indexes: the range of liver resection, duration of surgery, hepatic portal occlusion, intraoperative blood loss, and intraoperative blood transfusion were compared.
4. Hematological indexes: the ALT, AST, TBIL, DBIL, and albumin levels [15–18] at 1 day, 3 days, and 5 days after surgery were compared.
5. Postoperative recovery: the total amount of albumin infusion, postoperative ventilation time, postoperative hospitalization time, and hospitalization cost were compared.
6. Incidence of complications: complications included ascites, bile leakage, abdominal infection, and postoperative hemorrhage. The number of patients with complications was counted.

### 2.6. Statistical Treatment

In this study, the data were processed by SPSS 20.0 and graphed by GraphPad Prism 7 (GraphPad Software, San Diego, USA). This study included enumeration data and measurement data, tested by *X*^2^ and *t*-test. The difference was statistically significant when *P* < 0.05.

## 3. Results

### 3.1. Analysis of Patient Grouping

**Figure 1:**
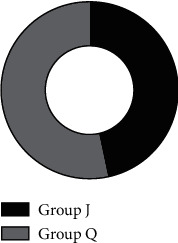
Analysis of patient grouping (*n*(%)). The black area represents group J (*n* = 28, 46.7%), and the gray area represents group Q (*n* = 32, 53.3%).

The grouping of patients is shown in Figure 1.

### 3.2. Comparison of Preoperative Situation

No obvious difference in preoperative situation was found between the two groups (*P* > 0.05), and the liver function indexes of all patients were within the normal range (Table 1).

### 3.3. Comparison of Surgery-Related Indexes

**Figure 2:**
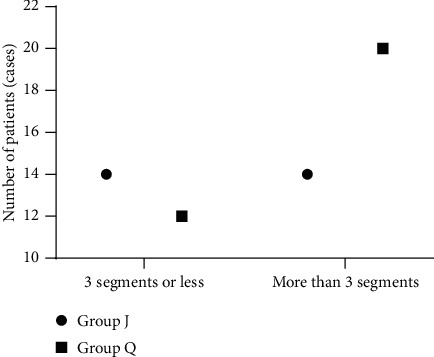
Scope of liver resection. The abscissa from left to right represent 3 segments or less and more than 3 segments, respectively, and the ordinate represents the number of patients (cases). The dots represent group J, and the squares represent group Q.

The duration of surgery in group J was notably shorter compared with group Q (*P* < 0.05; Figure 2 and Table 2).

Group J included 14 patients (50%) with a resection of 3 segments or less and 14 patients (50%) with a resection of more than 3 segments. Group Q included 12 patients (37.5%) with a resection of 3 segments or less and 20 patients (62.5%) with a resection of more than 3 segments. The comparison between the two groups showed *X*^2^ = 0.950 and *P*=0.330.

### 3.4. Comparison of Hematological Indexes

ALT, AST, DBIL, and albumin in group J were notably different from those of group Q (*P* < 0.001) at 1 day after surgery. ALT and AST in group J were notably different from those of group Q at 3 days after surgery (*P* < 0.001). No obvious differences were observed in the hematological indexes between the two groups at 5 days after surgery (*P* > 0.005) (see Table 3).

### 3.5. Comparison of Postoperative Recovery

The total amount of albumin infusion, postoperative hospitalization time, and hospitalization cost in group J were notably lower compared with group Q (*P* < 0.001; Table 4).

### 3.6. Comparison of the Incidence of Complications

**Figure 3:**
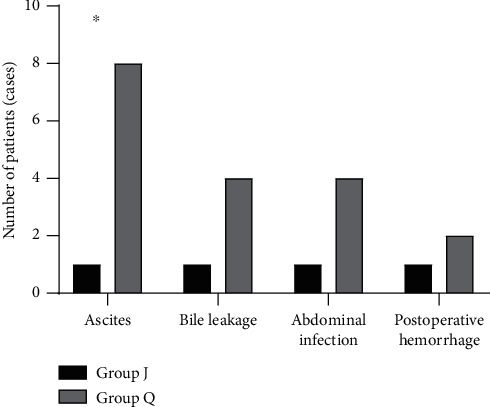
Comparison of the incidence of complications. The abscissa from left to right represent ascites, bile leakage, abdominal infection, and postoperative hemorrhage, and the ordinate represents the number of patients (cases). The black area represents group J, and the gray area represents group. Q ^*∗*^*P* < 0.05. Ascites occurred in 1 case of group J and 8 cases of group Q. Bile leakage occurred in 1 case of group J and 4 cases of group Q. Abdominal infection occurred in 1 case of group J and 4 cases of group Q. Postoperative hemorrhage occurred in 1 case of group J and 2 cases of group Q.

The incidence of complications in group J was notably lower compared with group Q (*P* < 0.05; Figure 3).

## 4. Discussion

With the continuous progress of relevant medical technology in recent years and significantly improved level of liver cancer surgery in China, how to optimize the prognosis of LC patients undergoing surgery by preoperative assessment has become the focus of clinical research. Complication with underlying liver diseases such as liver cirrhosis in LC patients in China can increase the damage to liver cells and affect liver nutritional and metabolic functions. At the same time, due to the decline of appetite in LC patients and reduced nutrition intake with different degrees of Warburg effect, the incidence of malnutrition in patients can reach 80%, adversely affecting the postoperative recovery of patients [19–21]. At present, the preoperative assessment of liver cancer patients includes liver function classification and hepatic functional reserve test, while nutritional assessment is not included. However, clinical practice has confirmed the key role of nutritional assessment, so close attention should be paid to the selection of appropriate preoperative nutritional assessment tools in doctors.

As a common preoperative nutritional assessment tool in clinical practice, NRS2002 is superior to other assessment scales in sensitivity, which can be adopted to screen nutritional risk indicators of hospitalized patients and is associated with the prognosis of patients [22, 23]. NRS2002 can be used in the preoperative assessment of various diseases. In this study, it was applied to the preoperative nutritional assessment of LC patients, and 60 patients were split into group J (*n* = 28) without nutritional risk and group Q (*n* = 32) with nutritional risk according to the classification of NRS2002. The two groups showed no obvious difference in preoperative situation, and patients' liver function indexes were within the normal range, which could be used for study.

Anesthesia, trauma, and other factors during liver cancer surgery can cause high catabolism. If patients have poor preoperative nutritional status and low surgical tolerance, their perioperative body consumption will be higher than that of ordinary patients, further increasing the possibility of metabolic disorders after surgery and seriously affecting the recovery during anesthesia. Moreover, liver cancer surgery will lead to serious damage to liver function. Malnutrition will further aggravate liver damage, significantly reduce the frequency of albumin synthesis, and increase the incidence of complications such as hypoproteinemia, infection, and poor healing, thus slowing the recovery of patients after surgery. This study showed that the duration of surgery and postoperative hospitalization time in group J were notably shorter compared with group Q (*P* < 0.05), indicating that malnutrition will worsen the body condition of patients and increase the surgical risk. ALT, AST, DBIL, and albumin in group J were notably different compared with group Q (*P* < 0.001) at 1 day after surgery. ALT and AST in group J were notably different compared with group Q at 3 days after surgery (*P* < 0.001). The above results indicated that patients with malnutrition had more serious liver damage, with unsatisfactory postoperative recovery.

The study also found that the hospitalization cost in group J was notably lower compared with group Q (*P* < 0.001), which may be related to the higher incidence of complications in group Q. Group Q received more albumin infusion, resulting in an obvious increase of the hospitalization cost. This study also showed that patients with malnutrition were more likely to have complications such as ascites and bile leakage compared with group J, but no serious complications or death occurred. This is because the patients in this study received comprehensive evaluation and perioperative management before surgery, as well as enteral nutrition support after surgery. Therefore, both groups of patients had certain recovery.

According to the study of Debanjan et al., the albumin infusion volume of patients with malnutrition undergoing liver cancer surgery was (41.65 ± 13.57) g, which was significantly higher than that of patients with normal nutrition (*P* < 0.001) [24], revealing that malnutrition can affect the body state and aggravate the perioperative consumption of patients. Therefore, it is of great importance to deepen the study on chronic nutritional consumption of such patients.

In conclusion, liver cancer is a common malignant disease affecting life and health of Chinese residents. This study has confirmed that postoperative recovery of LC patients is closely related to their preoperative nutritional status, and those with poor nutritional status have a high incidence of postoperative complications and long recovery time. NRS2002 can effectively screen the nutritional status of patients and provide reference for prognosis evaluation and the application of nutritional support programs.

## Figures and Tables

**Table 1:** Comparison of preoperative situation.

Group	Group J (*n* = 28)	Group Q (*n* = 32)	*X*^2^/*t*	*P*
Gender			0.234	0.628
Male	14 (50.0%)	14 (43.8%)		
Female	14 (50.0%)	18 (56.3%)		

Age (years old)				
Range	32–74	33–74		
Average age	51.21 ± 6.20	51.23 ± 6.21	0.012	0.990

Underlying diseases				
Diabetes	6 (21.4%)	10 (31.3%)	0.737	0.391
Hypertension	3 (10.7%)	6 (18.8%)	0.756	0.384
ALT (U/L)	38.98 ± 9.12	42.11 ± 9.65	1.286	0.204
AST (U/L)	30.15 ± 4.65	32.15 ± 5.36	1.533	0.131
TBIL (*μ*mol/L)	14.99 ± 0.54	15.10 ± 0.65	0.707	0.482
DBIL (*μ*mol/L)	4.10 ± 0.35	4.24 ± 0.65	1.017	0.313
Albumin (g/L)	40.10 ± 0.68	39.98 ± 1.54	0.381	0.705

**Table 2:** Comparison of surgery-related indexes.

Items	Group J (*n* = 28)	Group Q (*n* = 32)	*X*^2^/*t*	*P*
Duration of surgery (min)	215.68 ± 26.87	365.98 ± 35.15	18.399	<0.001

Hepatic portal occlusion			0.950	0.330
Yes	14 (50.0%)	20 (62.5%)		
No	14 (50.0%)	12 (37.5%)		

Intraoperative blood loss (ml)	295.12 ± 60.98	565.98 ± 48.45	19.156	<0.001

Intraoperative blood transfusion			2.402	0.121
Yes	4 (14.3%)	10 (31.3%)		
No	24 (85.7%)	22 (68.8%)		

**Table 3:** Comparison of hematological indexes ($\bar{x}\pm s$).

Items	Group J (*n* = 28)	Group Q (*n* = 32)	*t*	*P*
1 day after surgery				
ALT (U/L)	205.65 ± 28.54	489.35 ± 65.12	21.314	<0.001
AST (U/L)	180.54 ± 25.48	470.65 ± 98.54	15.128	<0.001
TBIL (*μ*mol/L)	20.99 ± 2.54	22.65 ± 5.21	1.533	0.131
DBIL (*μ*mol/L)	6.10 ± 0.54	13.54 ± 3.68	10.588	<0.001
Albumin (g/L)	32.11 ± 0.65	30.98 ± 1.20	4.442	<0.001

3 days after surgery				
ALT (U/L)	178.65 ± 24.65	356.98 ± 98.52	9.317	<0.001
AST (U/L)	75.45 ± 10.58	210.65 ± 92.12	7.714	<0.001
TBIL (*μ*mol/L)	22.65 ± 2.54	23.65 ± 2.14	1.655	0.103
DBIL (*μ*mol/L)	8.21 ± 1.68	9.11 ± 2.15	1.788	0.079
Albumin (g/L)	34.12 ± 1.65	33.68 ± 0.65	1.391	0.169

5 days after surgery				
ALT (U/L)	120.65 ± 25.65	125.98 ± 26.98	0.781	0.438
AST (U/L)	46.98 ± 4.25	49.11 ± 5.87	1.589	0.117
TBIL (*μ*mol/L)	20.12 ± 2.35	20.98 ± 2.68	1.313	0.195
DBIL (*μ*mol/L)	7.10 ± 1.24	7.65 ± 1.64	1.448	0.153
Albumin (g/L)	35.98 ± 0.64	35.87 ± 0.98	0.507	0.614

**Table 4:** Comparison of postoperative recovery ($\bar{x}\pm s$).

Items	Group J (*n* = 28)	Group Q (*n* = 32)	*t*	*P*
Total amount of albumin infusion (g)	13.10 ± 3.48	41.65 ± 13.57	10.815	<0.001
Postoperative ventilation time (d)	2.56 ± 0.35	2.68 ± 0.35	1.325	0.190
Postoperative	9.25 ± 0.30	13.11 ± 0.58	31.680	<0.001

## Data Availability

All data can be provided by the corresponding author upon request.
